# Supplementary material for: Are carbon emissions trading and green financial instruments synergistic? -Comprehensive quantitative research based on content analysis
Source: PLoS One. 2024 Mar 7;19(3):e0298601. doi: 10.1371/journal.pone.0298601 (PMC10919601; doi:10.1371/journal.pone.0298601)
Supplement: S1 Data — (ZIP) [file pone.0298601.s001.zip › Data/地级市Z_F工作报告.zip!/╣≤╓▌/░▓╦│╩╨/└┤╫╘╣1⁄2╓┌║┼í╛┬φ┐╦╩2╛▌═°í┐_2010.docx]

各位代表:

现在，我代表市人民政府，向大会作工作报告，请予审议，并请各位政协委员和其他列席人员提出意见。

2009年政府工作回顾

2009年，面对国际金融危机的严重冲击，在中共安顺市委的正确领导下，在市人大、市政协的监督、支持和帮助下，我们坚持以邓小平理论和“三个代表”重要思想为指导，深入学习实践科学发展观，把扩内需、保增长作为首要任务，抢抓机遇，迎难而上，加快发展，大力推进新型工业化、城镇化、旅游产业化、农业现代化和优化发展环境工作，努力克服前进中的各种困难和问题，经济社会实现平稳较快发展。

2009年全市实现生产总值180.41亿元，同比增长12.0%;财政总收入首次突破30亿元，达到32.87亿元，同比增长24.5%，地方一般预算收入16.19亿元，同比增长16.8%;全社会固定资产投资84.24亿元，同比增长23.2%，纳入地方统计的固定资产投资72.19亿元，同比增长31.4%;社会消费品零售总额59.09亿元，同比增长19.7%;城镇居民人均可支配收入13327元，实际增长8.0%;农民人均纯收入3110元，实际增长8.8%;年末全市金融机构存、贷款余额分别为277.89亿元和204.63亿元，比年初增加66亿元和46亿元。

一年来，我们主要做了以下工作:

(一)狠抓中央扩内需、保增长项目的实施，发展基础进一步夯实

新增中央投资项目全面实施。我们紧紧抓住中央扩内需、保增长的政策机遇，在重大基础设施和保障性住房、农村民生工程、社会事业发展、节能减排和生态建设等方面，争取到四批新增中央投资项目332个，总投资12.03亿元，实现了项目百分之百开工，配套资金百分之百到位，整改要求百分之百落实，有力地拉动了经济增长。

以交通、水利为重点的基础设施建设全面推进。普定至安顺及安顺西绕城高速公路，惠水经紫云至兴仁、六枝至大山高速公路工程开工。镇胜高速公路坝陵河大桥建成通车。厦蓉高速公路平坝段建设前期准备工作基本结束。完成通乡油路163公里，通村公路500公里，通村油路41公里。长昆快速铁路安顺段三个站点选址工作完成。坝草码头改扩建工程顺利推进。黔中水利枢纽一期工程顺利开工，董箐电站合闸蓄水，鲁嘎水库、油菜河水库等“滋黔”工程和安西灌区续建配套与节水改造工程进展顺利。完成16座病险水库除险加固工程和8.5万亩烟水配套工程。黄家湾水利枢纽项目前期工作加快推进。

(二)狠抓产业结构调整，经济发展的质量和效益不断提高

农业基础地位进一步巩固。粮食总产量达81.11万吨，同比增长0.8%，连续七年创历史新高。实现农林牧渔业总产值54.90亿元，同比增长6.0%，畜牧业产值占农林牧渔业总产值比重达42.0%。把茶产业作为农业结构调整的重要内容，新增茶园3万亩，茶叶种植面积达10.45万亩。果蔬、优质米、油菜、烤烟和中药材等地方优质产业加快发展。减少农村贫困人口3.86万人，异地扶贫搬迁241户、1000人，解决农村饮水安全及饮水困难3.2万户、13.2万人。“家电下乡”实现销售总额5669万元，兑现财政补贴资金544万元。新农村建设扎实推进，23个省、市级新农村建设试点项目取得较好成效。

工业经济稳定增长。纳入地方统计的规模以上工业实现增加值39.55亿元，同比增长15.3%。煤电、建材等能源原材料工业，药品、食品、白酒等特色优势轻工业对工业经济的支撑作用进一步增强。原煤产量达998万吨，同比增长13.6%。发电量86.71亿千瓦时，同比增长11.7%。总投资50.88亿元的黄果树铝业、宏盛化工、超宇水泥、天马轴承、安酒技改以及黄瀑肉类加工等17个重点工业项目顺利推进。民用航空产业基地产业发展和空间布局规划获得批准。30座级通用飞机可研报告通过专家评审。入驻航空产业基地的重大项目总投资达27.3亿元。

以旅游为龙头的服务业加快发展。旅游基础设施建设加快推进，黄果树新城建设和半边街搬迁进展顺利，酒店改造建设步伐加快。屯堡文化旅游资源进一步整合，黄果树、龙宫景区接受了国家旅游局5A级景区复核。成功举办了黄果树瀑布节、央视“寻宝--走进安顺”等活动。到北京、杭州、青岛、广州、重庆、昆明和台湾等地开展旅游宣传推介活动。安顺荣获“2009年中国十大特色休闲城市”称号。在旅游业的带动下，交通运输、仓储物流、金融保险、酒店餐饮、邮电通信等第三产业迅速发展，实现增加值71.79亿元，同比增长12.1%。

(三)狠抓城市规划建设，城镇化进程进一步加快

规划编制工作成效明显。《安顺市城市总体规划(修编)》顺利通过省级评审。《安顺市土地利用总体规划(修编)》、《安顺市市域城镇体系规划》、《安顺市城市近期建设规划》、《安顺市城市道路网规划》等编制完成。启动了新火车站片区、杨家湖片区、北部新城等一批详规编制。各县区详规覆盖率达80%以上。

城市基础设施建设有力推进。先后完成了中华东路、东二环路等城区主干道路的改造和建设，对城区部分小街小巷进行了治理改造。完成城区30余条道路和7个节点绿化改造工程，改造绿地3万余平方米，城市绿化覆盖率达31%。完成城区液化天然气接收站建设。完成城乡电网建设投资9.31亿元，新增线路生产能力3250公里。市二中新校区建设进展顺利。启动市人民医院迁建工作。市殡仪服务中心、城市公交枢纽总站和城区站台等工程建设扎实推进。各县城基础设施建设成效明显，一批重点乡镇建设步伐加快。

城市管理进一步加强。按照“统一领导、分级管理、重心下移、区街为主”的思路，进一步明确了市与两城区的职责，初步形成“两级政府、三级管理、四级网络”的城市管理工作格局。加大征地拆迁和城区闲置土地清查力度，确保了城市建设顺利推进。深入开展“整脏治乱”专项行动和“与文明同行——满意在安顺”活动，进一步提升了城乡文明程度。

(四)狠抓生态文明建设，可持续发展的能力得到增强

生态建设和环境保护力度加大。退耕还林成果进一步巩固，完成营造林14.19万亩，全市森林覆盖率达37%。完成石漠化综合治理面积61.33平方公里、水土流失综合治理面积138.3平方公里，新增有效灌溉面积10.24万亩。启动虹山湖、娄家湖水环境治理工作。市污水处理厂正式运营，各县区污水处理厂相继建成并投入试运行，垃圾填埋场建设工程有序推进。新建农村沼气池21687户，“改厨、改厕、改圈”工程建设力度加大。节能减排工作取得实效，预计全市万元工业生产总值能耗同比下降4.63%，规模以上万元工业增加值能耗同比下降3.56%。化学需氧量和二氧化硫排放量同比分别下降11.28%和12.24%。城市空气质量优良率达100%。

(五)狠抓保障和改善民生各项工作，社会和谐稳定

社会保障体系建设进一步加强。新增城镇就业1.57万人，安排困难人员就业3475人，城镇登记失业率4.09%。认真落实再就业优惠政策，支出就业专项资金4300万元，减免各种税费170.93万元。将全市符合条件的对象全部纳入城乡低保覆盖范围，共发放低保金2.1亿元。城镇居民基本医疗保险全面推进。认真落实城乡医疗救助政策，共救助2.77万人，发放救助金1072.08万元。为15.8万名农村困难群众缴纳农村合作医疗参保费261.14万元。发放农村五保供养金773.85万元。启动了平坝县新型农村社会养老保险试点。

保障性安居工程顺利推进。完成农村危房改造16225户。建成经济适用住房1859套和廉租住房1272套。20个新增中央投资补助廉租住房项目全部开工建设。

各项社会事业协调发展。“两基”成果进一步巩固，全市小学适龄儿童入学率达99.3%，初中阶段毛入学率达103.2%。完成51所农村义务教育阶段薄弱学校改造工程。完成义务教育阶段学校实施绩效工资工作。中职招生突破万人大关。安顺学院、安顺职业技术学院办学条件进一步改善，办学水平进一步提高。启动医药卫生体制改革工作，全市新农合参合率达93.83%，人均筹资水平提高到100元。全面落实重大传染病和地方病防控工作措施，甲型H1N1流感得到及时有效防控，5万户地氟病防治炉灶改良任务顺利完成。新建和改扩建21个乡镇卫生院、9个乡镇计生服务站。全市人口出生率和自然增长率分别为13.0%。和6.2%。。新增普定县为国家级人口和计划生育优质服务先进县。完成第二次经济普查工作。文化体制改革取得明显成效，制定并实施安顺市文化产业发展规划，创作了《我的巴身小我的家》等一批优秀文艺作品。第三次文物普查工作取得阶段性成效，新增18个非物质文化遗产项目进入第三批省级保护名录和第一、二批省级扩展名录。王若飞故居被命名为国家级国防教育基地，故居陈列馆建成并免费开放。完成22个乡镇综合文化站、1686个文化信息资源共享工程、860个20户以上自然村广播电视村村通工程、74个农民体育健身工程。信访工作得到加强，社会治安综合治理力度加大，“平安安顺”建设扎实推进。生产安全事故起数和死亡人数实现“双降”。启动了创建全国文明城市、国家卫生城市和国家环保模范城市“三创”工作。双拥工作不断加强。国防建设与经济建设协调发展。科技和知识产权工作进一步加强，在全省率先整体进入省级科技进步市行列。人事人才、审计、统计、民族宗教、人防、老龄、地方志、档案、气象、对台、外事侨务等各项工作都取得了新的成绩。“十件实事”基本完成。

(六)狠抓体制机制创新，发展的内在活力得到增强

改革创新力度进一步加大。政府机构改革工作扎实推进。市属国有企业改制工作基本完成。集体林权制度主体改革全面完成。理顺了市与县区之间的财税分配关系。组建城市商业银行。市金融办和国资公司、城投公司、交投公司“一办三平台”投融资作用得到良好发挥。出台《关于大力推进非公有制经济又好又快发展的实施意见》等政策措施，成立市优化发展环境督查办公室和各县区中小企业(非公经济)服务中心，发展一批小额贷款公司，有力地推进了非公有制经济的发展。率先在全省开展非公有制企业职称评审工作。下大力整治企业周边环境，企业的生产条件得到改善。市县区属非公有制工业实现增加值22.69亿元，占市县区属工业增加值的89.7%，同比增长13.2%。

扩大开放取得积极成效。进一步加强与珠三角、长三角、西南六省区市、对口帮扶城市及友好合作城市的联系和交流。成立了市人民政府驻上海、广州联络处。与浙江金华缔结为友好合作城市。与国家开发银行签订了60亿元的合作框架协议，支持民用航空产业基地建设。与省建行签订了100亿元的合作框架协议，与省移动公司、省电信公司签订了战略合作框架协议。“九顺合作”深入推进，与农工党达成“农安合作”协议，与致公党开展交流合作。全年招商引资到位资金52.55亿元，完成目标任务的116.8%。

各位代表，过去的一年，我们把加强政府自身建设作为推动经济社会平稳较快发展的重要保障。坚持民主集中制原则，不断完善政府议事决策机制。自觉接受人大的法律监督、工作监督和政协的民主监督，共办理人大代表建议111件，政协提案193件，办复率100%，满意率99.7%。行政执法责任制和问责制落实力度不断加大。应急管理工作进一步加强。办公自动化建设取得新成效。政府系统党风廉政建设深入推进。与工会、共青团、妇联、文联、残联、红十字会、科协等人民团体的联系进一步加强。

回顾过去一年不平凡的历程，我们不仅在应对挑战中实现了保增长目标，而且在克服困难中增强了发展后劲，取得了超出预期的发展成果。这些成绩的取得，是中共安顺市委正确领导的结果，得益于市人大、市政协的监督和支持，凝聚着全市各族人民的心血和汗水。在此，我谨代表市人民政府，向全市工人、农民、知识分子、干部、离退休老同志，向各民主党派、工商联、无党派人士、人民团体，向驻安人民解放军指战员、武警官兵、公安政法干警，向关心和支持安顺建设与发展的社会各界人士，表示衷心的感谢并致以崇高的敬意!

在肯定成绩的同时，我们也清醒地看到，经济社会发展中仍然存在一些困难和问题，主要表现在:一是经济总量小，财政实力弱，经济抵御风险能力不强。二是农民增收渠道还不多，新阶段扶贫开发任务仍然十分繁重。三是城乡公共服务设施建设欠账较多，城镇化水平不高，城市聚集辐射能力弱。四是部分群众生活比较困难，保障和改善民生的力度还需继续加大。五是政府行政效能、管理服务水平与形势的要求和人民群众的期待还有一定差距，发展环境仍需进一步优化。对于这些困难和问题，我们将高度重视，认真研究，继续采取有效措施，切实加以解决。

2010年政府工作主要任务

今年政府工作的总体要求是:高举中国特色社会主义伟大旗帜，以邓小平理论和“三个代表”重要思想为指导，深入贯彻落实科学发展观，全面贯彻党的十七大和十七届三中、四中全会，中央、省经济工作会议精神，坚持解放思想，好字优先，能快则快，抢抓国家继续实施积极的财政政策、适度宽松的货币政策和深入推进西部大开发的机遇，着力优化发展环境，加快转变经济发展方式，坚定不移地推进工业化、城镇化、旅游产业化、农业现代化和信息化，切实保障改善民生、促进社会和谐，努力实现经济社会更好更快发展。

今年经济社会发展的主要预期目标是:生产总值增长10%以上;全社会固定资产投资增长18%以上;社会消费品零售总额增长15%;地方一般预算收入增长10%以上;城镇居民人均可支配收入实际增长7%，农民人均纯收入实际增长7%;城镇登记失业率控制在4.5%以内;人口自然增长率控制在7%。以内;单位生产总值能耗降低4.2%，化学需氧量和二氧化硫排放量分别下降7%和3.5%。

围绕上述总体要求和目标任务，今年要重点抓好以下八个方面的工作。

(一)以加强交通水利基础设施建设为抓手，不断改善发展的基础条件

加强交通路网建设。紧紧围绕我市率先在全省实现县县通高速的目标，完成普定至安顺及安顺西绕城高速公路建设的征地拆迁工作和路基工程量的60%，力争开工建设西秀至板当高速公路，积极配合做好六枝至大山高速公路及龙宫联络线、惠水经紫云至兴仁高速公路、厦蓉高速公路平坝段建设工作。做好镇宁至乐纪、织金至普定高速公路前期工作，力争早日开工建设。加快推进坡蝉至板贵公路提等改造工程。改造建设云马厂等5个军工企业的进厂公路。完成所有通乡油路、通村公路项目建设，在全省率先实现所有乡镇都有油路(水泥路)通达，所有建制村通公路。加快黄织铁路建设，配合做好黄桶经紫云至百色铁路建设前期工作，抓好长昆快速铁路安顺境内三个站点的规划建设工作。抓好安轿铁路专用线战略装车场及安顺市煤矿运输公路和铁路联运项目的相关工作。规划建设市长途汽车客运站和市交通枢纽组织中心，完成镇宁、关岭客运站建设，建成6个农村客运站、6个农村渡口。继续配合做好西南水运出海通道镇宁坝草码头的改造建设工作。

加强水利设施建设。全力以赴做好黔中水利枢纽一期工程涉及我市的征地拆迁、移民安置等工作。加快鲁嘎水库、油菜河水库等“滋黔”一期工程建设，积极做好“滋黔”二期工程和新的大型灌区项目申报工作，努力争取黄家湾水利枢纽、新场河水库及一批小型骨干水源工程立项。加快安西灌区节水续建配套和“三小”等工程建设进度。新增有效灌溉面积7.55万亩，完成16座病险水库除险加固工程，新增水土流失综合治理面积100平方公里。认真实施农村人口人均半亩基本口粮田工程。完成板贵乡引水工程。进一步科学合理开发利用地表水和地下水，大力推广以管代渠、长距离管道送水。新增解决15万农村人口饮水安全问题。

加强项目工作。围绕提高项目库建设能力、向上争取项目能力、招商引进项目能力、项目融资能力、项目实施管理能力，扎实做好项目工作。加大项目前期工作经费投入，建立项目风险评估和筛选机制，努力提高项目的成熟度和竞争力。坚持走市场化的道路，通过多种途径引导更多的社会投资参与项目建设，破除资金瓶颈。加强项目库建设和管理，形成“投产一批、续建一批、开工一批、储备一批”的良性互动机制。建立健全项目跟踪服务制度，完善重大项目领导联系责任制，切实提高获批项目的开工率和完工率。认真做好“十二五”规划编制工作，努力增强规划的针对性、科学性和可操作性，充分发挥规划指导经济社会发展的作用。

(二)以工业作为加快经济发展的突破口，大力实施工业强市战略

做大做强能源原材料工业。积极争取安电三期进入国家和省的规划。支持董菁电站、马马崖电站早日全面投产达产。按照“优进劣退”和集团化的原则，鼓励支持战略投资者加快煤炭资源开发和煤矿整合步伐，重点抓好普定肖家湾煤矿、平坝鑫源煤矿等一批骨干矿井的建设。启动南部矿区勘探工作。按照循环经济的理念，依托能源工业大力发展煤化工、煤电化、煤电铝一体化等工业，努力实现资源的经济效益本地最大化。积极争取实施用电大户与发电企业直接交易试点。支持黄果树铝业、宏盛化工、宏大铝业、昌兴水泥、超宇水泥、红星发展、宏泰化工、永诚铁合金等企业发展壮大。

加快提升装备制造业整体水平。继续支持民用飞机、高级教练机、飞机维修等航空产业项目的发展，引进一批民用航空产业配套项目，加快把安顺建设成为产业特色鲜明、竞争力强的航空产业基地。依托军工企业的技术优势，积极探索军民结合的市场化途径，促进军工企业与地方经济共同发展。进一步强化政策支持，加大协调服务力度，支持云马客车、莲花轿车、电动车、采棉机、轴承、石油机械、医疗机械等产品发展，扩大市场规模。

大力发展特色轻工业。利用百灵集团上市的机遇，提升民族制药产业整体实力。支持顺健、玖久、寿仙等企业发展。按照盘活存量、做大总量、提质增效的目标，振兴安顺白酒产业。实施好安酒年产5000吨技改项目，加快普定、平坝、镇宁等地白酒业发展。支持金星啤酒异地技改扩能项目的实施。加快推进烟叶薄片项目建设。扎实推进雨润集团黄瀑肉类加工项目建设，确保年内建成投产。加大蜡染、波波糖、傩雕面具、仿真古生物化石等富有地方特色的旅游商品研发生产力度，努力在扩大规模、提高产业发展水平上实现突破。

加强工业园区建设。加快工业园区基础设施建设，抓好园区道路、电网、给排水、通信、标准厂房等硬件建设，为企业入驻创造良好条件。按照“产业发展集群化、集群发展园区化”的思路，积极推动工业企业向园区聚集，形成规模效应。建立健全园区共建共享机制，支持发展“飞地”经济。

加大科技创新力度。推动以企业为主体、市场为导向、产学研相结合的技术创新体系建设，积极申报实施一批国家科技支撑计划和省重大科技专项。加强与上海市科委、省科技厅的合作，力争在重点领域和重点产业方面取得新的成效。加强知识产权创造、运用和保护。以信息网络基础设施和数据共享平台建设为重点，加快“数字安顺”建设，大力推进信息化和工业化的融合。

(三)以建设百万人口大城市为目标，全力推进城镇化进程

抓好规划的编制和组织实施。启动新一轮城区控制性详规的编制和修订工作，尽快实现控制性详规全覆盖。完成五个县域体系规划。完成建制镇总体规划的修编和编制工作。完成虹山湖片区、新火车站片区、杨家湖片区、滨水景观等规划的编制。启动城市综合交通规划编制。加强城市重要节点的规划设计，提升城市品位。超前做好城市轻轨规划工作。发挥好规划在城市建设中的龙头作用，以规划指导和引领城市建设，切实维护规划的权威性和严肃性。

加快城市改造建设。根据新修编的城市总规，以北部新城、新火车站片区、航空产业基地、西秀新区和黄果树大街“四片区一轴线”为近期城市建设的重点区域，掀起新一轮城市建设高潮。抓好中华西路片区、建新菜场片区旧城改造和管元村、汪家山村“城中村”改造等项目的实施。推进儒林路等历史文化街区的保护开发。加快体育路、龙青路建设步伐，确保年内实现通车。完成西航路、两六路两所屯至青山段改造工程。完成中华西路、市西北路主体工程，开工建设武当路、北山路、安普路等工程，尽快形成中心城市道路骨架网络。继续推进小街小巷综合整治。实施城区人行天桥和地下通道建设，完成老大十字地下人防工程，在重要节点实现人车分流。完成若飞公园建设。继续实施城区电网改造。抓好“引千入虹”和污水处理厂中水回用工程建设。启动开发区国际旅游商贸会展中心建设。抓好市医院、行政学院、安顺职业技术学院迁建工作，加快推进市二中新校区建设。开工建设城区垃圾收运系统、贯城河西支流改造和航空产业基地污水、供水管网改扩建等工程。启动桂家湖水厂、安顺供水二期等项目前期工作。

加强城市管理。进一步巩固“两级政府、三级管理、四级网络”城市管理工作格局。抓好城市管理相对集中处罚权实施工作，理顺和规范市区两级拆违机制，严肃查处违法建设。加大闲置土地清理力度，杜绝囤地、圈地行为。完善市与两城区权责一致、利益共享的城市开发建设机制，形成共同推进城市建设的合力。继续推进“整脏治乱”专项行动和“与文明同行——满意在安顺”活动，大力实施城市精细化管理和城市亮化、美化、绿化、净化工程。

推进城镇化进程。认真落实土地利用总体规划对城市建设用地的安排，切实保障用地需要。高度重视城市新区开发中的配套设施建设和商业培育，通过商业发展积累财富，形成城市投资、建设、发展的良性循环。依托快速铁路、高速公路及其场站建设，做好沿线城镇产业发展规划，尽快培育一批各具特色的产业区和产业带，推进城镇发展。积极支持各县城与重点城镇的基础设施和环境建设，把公共服务设施向农村延伸。坚持节约集约用地，努力提高城镇现有土地利用率。按照“一年大见成效，两年全面完成”的要求，加大境内高速公路沿线景观整治力度，形成具有黔中地域文化特色的建筑景观。加快把在城市中有相对固定住所和相对固定工作的本市籍农民工特别是新生代农民工转变为城市居民。充分依托我市良好的生态气候优势，大力实施“气候移民”，扩大城镇人口规模。

(四)以推进旅游产业化为重点，大力发展服务业

加大旅游基础设施建设力度。做好屯堡文化景区旅游发展规划编制工作，进一步搞好屯堡旅游资源的保护和深度开发。做好花江大峡谷、坝陵河大桥等景区景点规划工作。开工建设天星桥、郎弓、木城河、花江大峡谷景区公路连线，构建大黄果树旅游经济圈公路环线。全力推进黄果树新城建设，完成半边街搬迁工作。改造建设虹山宾馆、燕安酒店、黄果树宾馆、佳缘国际大酒店等一批宾馆酒店，进一步提高旅游接待能力。引进和建设一批管理国际化、标准化的高档休闲娱乐项目。

加快旅游产品开发。进一步加大黄果树、龙宫、格凸河等景区的开发建设力度。做好关岭古生物化石群国家地质公园世行贷款项目建设工作。支持天龙、云峰八寨、讲义村、巴身小等一批乡村旅游景点加快发展。认真搞好旅游线路的设计，重点推出一批新的二日游、三日游精品旅游线路。采用市场化运作方式，继续办好黄果树瀑布节等旅游文化节庆活动。充分利用上海世博会的机遇，组织一系列主题突出、特色鲜明的旅游宣传促销活动，提高我市的知名度和美誉度。

大力发展现代服务业。抓好物流园区规划，做好国家物流示范园项目申报工作。积极培育和发展一批具有较强市场竞争力的商贸物流企业和大型运输企业，推进物流配送现代化。大力推进金融业发展，加快金融业与地方经济的融合，提高金融业服务地方经济社会发展的水平。认真落实国家促进房地产市场发展的各项政策措施，鼓励普通商品住房消费，稳定房地产投资和消费预期，推进我市房地产市场健康发展。大力发展市政公用事业、商贸餐饮、文化娱乐、健身养生、社区服务等面向民生的服务业。

(五)以促进农民增收为核心，扎实做好“三农”工作

巩固农业发展基础。认真贯彻落实中央一号文件精神，按照稳粮保供给、增收惠民生、改革促统筹、强基增后劲的基本思路，加大统筹城乡发展力度，进一步夯实农业农村发展基础。认真落实中央各项强农惠农政策，确保按照总量持续增加、比例稳步提高的要求，进一步增加“三农”投入，确保各项涉农补贴资金及时足额发放到农民手中。继续抓好以县为主财政支农资金整合试点。开展化解农村公益性债务试点工作，扩大农村公益事业“一事一议”财政奖补试点范围。支持农村金融机构建设，扩大农村基础金融服务覆盖面。全力抓好粮食生产，继续组织实施好75万亩粮食增产工程和粮油高产创建等重点项目。积极推进农业机械化。大力发展生猪和蛋鸡养殖，继续实施好草地生态畜牧业项目，扶持特色养殖业发展，提高畜牧业在农业中的比重。稳定烤烟生产，切实做好现代烟草农业示范工程，推进茶叶、果蔬、中药材等规模化发展，提高经济作物在种植业中的比重。

推进农业产业化。积极培育和引进农业产业化经营龙头企业，加快发展特色农产品加工业。大力扶持农民专业合作经济组织，完善企业与农民之间的利益联结机制。加强农产品市场信息服务、农业社会化服务等体系建设，积极支持农村物流业发展，继续推进“万村千乡市场工程”、“双百市场工程”建设，提高农产品商品率。高度重视农产品质量安全工作，建立健全市县乡三级农产品质量安全体系，推行标准化生产，加强农产品认证工作。加大农特产品商标和地理标志申请保护力度，积极培育和壮大一批名优品牌，提高农产品市场竞争力。

深入实施新阶段扶贫开发。实施产业化扶贫，改变传统的扶贫资金安排方式，集中用于培育典型、树立样板，带动农民增收致富。减少农村贫困人口3.09万人，完成异地扶贫搬迁307户、1500人。组织实施好“阳光工程”和“雨露计划”，大力推动对外劳务输出。认真落实“家电下乡”、“汽车摩托车下乡”和汽车、家电以旧换新等政策措施。扎实抓好社会主义新农村建设，结合发展乡村游、农家乐等多种途径，引导社会资金投向农村基础设施建设，促进农村面貌和人居环境不断改善。

认真做好抗旱救灾工作。针对去年秋冬以来严峻的旱情，切实做好当前抗旱保供水和抗旱保春耕工作，把抗旱保供水和抗旱保春耕工作作为当前关注民生、解决老百姓实际困难的一项重要工作来抓，从财力、物力、技术指导等方面及时解决抗旱中存在的实际困难，最大限度地满足当前群众生活用水和春耕生产用水的需要。

(六)以加强环境保护和生态建设为着力点，加快构建资源节约型和环境友好型社会

加强环境保护。扎实抓好石漠化综合治理试点工作，完成石漠化综合治理110平方公里。认真落实国家完善退耕还林政策措施，继续推进天然林保护、珠江防护林等林业重点工程建设，完成营造林14万亩，力争森林覆盖率提高一个百分点。认真抓好沼气工程建设，着力提高工程利用率。全面完成各县城垃圾填埋场建设。强化重点旅游景区及周边区域生态建设和城区绿化建设品质。

加强节能减排。严格环境准入，积极推动循环经济和环保产业项目发展，突出抓好重点行业、重点企业的节能管理和技术改造。加快用高新技术改造提升传统产业，逐步淘汰落后生产能力，形成有利于资源节约和环境保护的产业体系。加强矿山环境恢复保证金的使用和监管，有效保护和治理矿山生态环境。加强以虹山湖、娄家湖为重点的城市景观水环境综合治理。严格执行饮用水源保护区管理规定，强化集中式饮用水源地环境保护，确保水质全面达标。加强农业面源污染治理。继续保持城市空气质量达到国家二级以上标准。

(七)以保障改善民生为出发点和落脚点，加快和谐社会建设

认真做好就业和社会保障工作。实施更加积极的就业政策，加强对农民工和下岗失业人员的职业技能培训，积极引导和鼓励高校毕业生到基层和中小企业就业，努力开发公益性岗位，加大对就业困难人员、零就业家庭就业援助力度，力争城镇新增就业12800人。大力支持返乡农民工创业。加快完善社会保障体系建设，做好失业和工伤保险市级统筹工作，稳步推进医疗、生育保险市级统筹试点。做好参保人员养老关系转移接续工作。支持平坝县做好农村养老保险试点。继续做好城镇职工、城镇居民基本医疗保险工作。完善新型农村合作医疗制度，人均补助标准从80元提高到120元。落实好农村低保“提标扩面"政策。积极发展社会福利、慈善、救灾救济等事业。全力抓好以农村危房改造为重点的保障性安居工程建设，完成20430户农村危房改造任务。坚持租售并举，完成2858套廉租住房建设。进一步发挥住房公积金在住房保障体系中的作用。

全面发展各项社会事业。促进义务教育均衡发展。全面推进素质教育。巩固提高“两基”工作成果，确保紫云自治县顺利通过省政府“两基”复查和“普实”验收。抓好义务教育学校标准化建设，继续实施中小学校舍安全工程和农村义务教育阶段薄弱学校改造工程。健全义务教育经费保障机制，确保经济困难家庭子女、进城务工人员子女、农村留守儿童平等接受义务教育。抓好高中阶段教育发展规划编制工作。建立健全各级各类教育学生资助政策体系。加快县级职教中心建设，支持安顺职中创建国家级示范性中等职业学校。支持安顺学院提升办学水平和层次，支持安顺职业技术学院创建省级示范性高等职业技术院校。深化医药卫生体制改革，加快基本公共卫生服务均等化进程。落实国家基本药物制度，切实减轻群众基本药物费用负担。抓好县级医院、乡镇卫生院和村卫生室标准化建设。改革基层医疗卫生机构补偿机制和运行机制，逐步建立分级诊疗和双向转诊制度，为群众提供安全、有效、方便、价廉的基本医疗卫生服务。强化疾病防控、食品药品和卫生监督执法，高度重视重大传染病、地方病的防控工作。大力发展中医药事业。继续稳定和降低生育水平，统筹解决人口问题，推进人口计生“创优”工作。做好第六次人口普查工作。抓好人力资源开发和人才培养。重视发展妇女儿童、老龄、残疾人、红十字和关心下一代事业，继续做好外事、侨务、对台、民族宗教、气象、地方志、档案等工作。

加强社会主义精神文明建设。全面开展全国文明城市、国家卫生城市、国家环保模范城市“三创”工作，塑造安顺新形象。广泛开展群众性精神文明创建活动，不断加强社会主义核心价值体系和公民道德建设。加快县区图书馆、文化馆、档案馆和乡镇综合文化站、村(社区)文化室等基层文化设施建设，逐步建立覆盖城乡的公共文化服务体系。全面完成5910个20户以上自然村广播电视村村通工程。弘扬黔中文化，加快推进文化产业大发展、大繁荣。深化文化体制改革，全面完成经营性文化事业单位转企改制工作。加大物质和非物质文化遗产的保护利用力度。积极发展新闻出版和广播影视事业。加快公共体育设施建设，广泛开展全民健身运动。大力推进殡葬改革，合理规划建设墓地园区，倡导文明殡葬。不断加强国防后备力量建设，积极开展创建“双拥”模范城活动。

全力维护社会稳定。加强社会治安综合治理，切实推进社会矛盾化解、社会管理创新、公正廉洁执法三项重点工作，促进“平安安顺”创建活动深入开展，增强人民群众的安全感。做好新形势下的信访工作。进一步加强应急管理，提高应对处置突发公共事件和防灾减灾的能力。加强安全生产管理，健全生产安全监控体系，保持安全生产形势持续稳定。高度重视隐蔽战线工作，切实维护国家安全。

(八)以深化改革开放为强大动力，不断增强发展的内在活力

进一步推进体制机制改革创新。继续探索和深化试验区各项改革，努力把安顺建设成为贵州加快发展的经济特区。认真落实市委二届八次全会关于促进非公有制经济发展的政策措施，大力优化发展环境，培育壮大非公有制经济。扎实做好国有企业改革改制工作。加强国有资产监管和营运，确保国有资产保值增值。深化财税体制改革，着力培育壮大财源，不断增强财政实力。畅通政银企沟通渠道，鼓励引导金融机构加大对重点产业、重点项目信贷资金投放。认真落实国家资源性产品价格、税费等各项改革政策。基本完成事业单位岗位设置，全面推行事业单位人员聘用制度。进一步深化行政审批制度改革。继续深化农村综合改革，积极探索农村集体建设用地流转的有效途径。加快集体林权制度配套改革步伐。

进一步扩大开放。实行以商招商、企业招商、网上招商等灵活多样的招商方式，加大招商引资力度。以思想的大解放、观念的大更新进行招商，以海纳百川的胸襟、开放包容的心态进行招商，让投资者获利，让安顺发展。坚持招商与选商并重，杜绝高污染的企业进入，保住青山绿水，保护好生态环境。坚持引资与引智并重，借助外脑、借助外力推动安顺发展。紧紧抓住国家制定中西部地区承接东部产业转移政策的机遇，做好产业承接工作。发挥好驻北京和上海、广州联络处的作用，切实增强对外开放的针对性和实效性。推进贵阳城市经济圈建设，谋划好安顺与贵阳两地连接区域的发展规划，与贵阳实现同城化发展。积极做好保税区申报工作，为我市经济发展培育新的增长极。深入开展“九顺合作”、“农安合作”，进一步争取中央和省各民主党派的支持。

切实加强政府自身建设

新时期、新形势，对政府自身建设提出了新的更高要求。我们将以政府机构改革为契机，大力加强政府自身建设，进一步理顺和规范部门职能，不断提高政府工作水平，努力建设人民满意的政府。

第一，切实加强民主政治建设，不断提高政府工作的科学性。进一步健全民主决策制度，自觉接受人大的法律监督、工作监督和政协的民主监督，认真办理人大代表建议和政协建议案、提案。自觉接受人民法院和人民检察院的司法监督。接受新闻舆论和人民群众的监督。认真听取民主党派、工商联、无党派人士的意见和建议，支持工会、共青团、妇联等人民团体依法依章履行职责。支持监察、审计部门依法独立履行监督职责。进一步扩大基层民主，健全基层自治组织和民主管理制度，让广大群众更好地参与管理基层公共事务。大力推行政务公开，进一步提高政府工作的透明度。坚持民主集中制，完善重大问题集体决策制度、专家咨询制度、社会公示和听证制度，集中各方智慧和力量促进政府工作水平不断提高。

第二，切实推进依法行政，不断强化政府工作的规范性。增强依法行政意识，提高依法行政能力，自觉运用法律手段调节经济和管理社会事务，严格依照法定权限和程序行使权力、履行职责，使行政管理更加民主化、科学化、法制化。加强行政执法、法制教育等工作，进一步明确执法权限，减少执法层级，完善执法程序，提高执法水平。认真做好行政复议、行政应诉工作。加强对政府工作人员和执法人员的监督管理，对执法不公、越权执法、执法犯法、徇私枉法等行为严查严办。

第三，切实改进工作作风，不断增强落实力和执行力。进一步强化宗旨意识、大局意识、服务意识和效率意识，不断改进工作作风。进一步建立健全严格的工作责任制，切实解决“谁去执行、何时执行、执行什么”的问题。围绕中心工作量化目标任务，加大问责力度，对重点工作已经落实的问效果，正在落实的看进度，尚未落实的查原因，落实不力的追责任，让干与不干的不一样，干好与干坏的不一样。凡是定下来的事，就一定要想方设法、创造条件把它干好、干出成效，不达目的不罢休。

第四，切实加强党风廉政建设，不断促进政府工作廉洁高效。严格执行党风廉政建设责任制，按照建立健全惩治和预防腐败体系工作规划要求，认真落实党员干部”不愿腐败、不易腐败、不能腐败、不敢腐败”的惩防机制，深入推进政府系统党风廉政建设。认真贯彻落实党员领导干部廉洁从政若干准则，进一步规范政府系统党员领导干部从政行为。加快行政审批电子监察系统建设，营造良好的政务环境。坚持勤俭行政，反对铺张浪费，不断降低行政成本。解决好反腐倡廉建设中人民群众反映强烈的突出问题，坚决纠正损害群众利益的不正之风。继续加大对新增中央投资项目和涉及民生项目的监督检查力度，深入推进工程建设领域突出问题专项治理，实现项目建设进度、质量、安全和廉洁的统一。

各位代表，今年是“十一五”规划的最后一年，也是我市建市十周年，新的起点，新的征程，新的目标，催人奋进。让我们高举中国特色社会主义伟大旗帜，以邓小平理论和“三个代表”重要思想为指导，继续深入贯彻落实科学发展观，在中共安顺市委的坚强领导下，在市人大、市政协的监督和支持下，坚定信心、同心协力，开拓创新、奋勇拼搏，为实现全市经济社会发展历史性跨越、推进富民兴安宏伟大业而努力奋斗!
